# Demographic drivers of decline and recovery in an Afro-Palaearctic migratory bird population

**DOI:** 10.1098/rspb.2016.1387

**Published:** 2016-11-16

**Authors:** Catriona A. Morrison, Robert A. Robinson, Simon J. Butler, Jacquie A. Clark, Jennifer A. Gill

**Affiliations:** 1 School of Biological Sciences, University of East Anglia, Norwich Research Park, Norwich NR4 7TJ, UK; 2 BTO, The Nunnery, Thetford IP24 2PU, UK

**Keywords:** population dynamics, integrated population models, carry-over effects, migration, demography

## Abstract

Across Europe, rapid population declines are ongoing in many Afro-Palaearctic migratory bird species, but the development of appropriate conservation actions across such large migratory ranges is severely constrained by lack of understanding of the demographic drivers of these declines. By constructing regional integrated population models (IPMs) for one of the suite of migratory species that is declining in the southeast of Britain but increasing in the northwest, we show that, while annual population growth rates in both regions vary with adult survival, the divergent regional trajectories are primarily a consequence of differences in productivity. Between 1994 and 2012, annual survival and productivity rates ranged over similar levels in both regions, but high productivity rates were rarer in the declining southeast population and never coincided with high survival rates. By contrast, population growth in the northwest was fuelled by several years in which higher productivity coincided with high survival rates. Simulated population trajectories suggest that realistic improvements in productivity could have reversed the decline (i.e. recovery of the population index to more than or equal to 1) in the southeast. Consequently, actions to improve productivity on European breeding grounds are likely to be a more fruitful and achievable means of reversing migrant declines than actions to improve survival on breeding, passage or sub-Saharan wintering grounds.

## 1. Background

Understanding the demographic processes driving changes in population size is fundamental to identifying appropriate actions to recover declining populations [1,2]. Across Europe, severe population declines are currently being reported in a wide range of Afro-Palaearctic migratory bird species [3–5]. These declines are generally more severe in long-distance (trans-Saharan) than short-distance (within-Europe/North Africa) migrants [4–7] and are particularly evident in those species travelling to the humid tropics of sub-Saharan Africa [5,8]. This has led to suggestions that migrant declines are being driven by changes on wintering and passage sites [5,7]. However, declines are not occurring everywhere; for example, within Britain, examination of within-species variation in population trends has revealed substantially greater declines in English than Scottish breeding populations [9,10]. These regional differences are also evident in populations of resident species, strongly suggesting that breeding season conditions are contributing to the observed population trends [10]. Exploration of the demographic processes underlying these patterns is therefore urgently needed to aid the design of appropriate conservation actions, and to avoid potentially costly actions that may not be effective.

Identifying environmental drivers of population trends in migratory species is notoriously complex because of the range of conditions, which individuals can experience across the migratory range [11], and because environmental conditions can influence demographic rates both directly and in subsequent seasons [12–14]. For example, productivity can be influenced by local environmental conditions during the breeding season and/or carry-over effects of conditions experienced in distant non-breeding locations [15,16]. Environmental drivers of demographic trends may also not be the most appropriate focus of actions to alter those trends. For example, prolonged droughts in the Sahel region in the 1980s were associated with sharp increases in mortality of several Afro-Palaearctic migratory bird species [17–19], but conservation actions can do little to directly influence Sahelian rainfall patterns.

In recent decades, the large body of research into the severe declines in farmland birds across Europe has provided a framework for addressing population declines in widespread species; firstly, quantifying the extent of declines [20] and identifying the likely demographic and environmental drivers [21–23] before trialling bespoke management options [24] and policy mechanisms. For Afro-Palaearctic migrants, the magnitude of the population declines is clear and there is now an urgent need to understand the demographic drivers of these trends, and the feasibility of achieving sufficient demographic change to reverse these trends.

The relative influence of demographic rates on population trends can be explored using integrated population models (IPMs, [25,26]). IPMs simultaneously estimate trajectories of population size and demographic parameters by combining time-series of population abundance and key demographic rates, such as survival, fecundity or dispersal [27]. IPMs can also incorporate the influence of unmeasured demographic processes into population models, allowing their influence on population change to be explored [2,28,29].

In order to identify the demographic changes contributing to migratory bird population trends, we construct regional IPMs from national-scale surveys of productivity and survival rates for one of the suite of migratory species with regionally divergent population trends across Britain (willow warbler, *Phylloscopus trochilus* [30,31]), in order to quantify: (i) the relative contribution of survival and productivity to the divergent population trends, (ii) the demographic conditions that led to periods of population decline and recovery, and (iii) the demographic rates that would have been needed to reverse the population declines.

## 2. Material and methods

### (a) Regional integrated population models

Region-scale IPMs were constructed for a model migratory species with sufficient demographic data in each region (willow warbler); one for the northwest region in which populations are stable/increasing, and one for the southeast region in which populations are declining (see the electronic supplementary material, figure S1 and S1.1 for explanation of regional categorization). For each region, we modelled annual population growth rates (*λ_t_* = *N_t_*/*N_t_*_−1_) as a function of survival and recruitment using the IPM framework of [2], and count and demographic data collected in UK-wide surveys from 1994 to 2012; the period spanning the recent regional divergence in population trends (details below). Below we outline the modelling process, with full details and R code provided in the electronic supplementary material.

### (b) The population model

Birds have a multi-year life cycle, in which the population (*N*) in any given year (*t*) comprises adult individuals (*N*_a_) surviving from previous years and recruits (*N*_r_) hatched the previous year:2.1
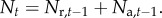


We estimate *N_t_* using counts from line-transect surveys collected in a stratified random sample of 1 km squares in each region ([32]; electronic supplementary material, S1.1).

Recruitment and survival are typically stochastic and may be characterized by Poisson and binomial processes, respectively:2.2
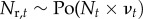
and2.3
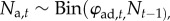
where *ν_t_* represents the mean number of young per breeding attempt recruiting into the adult population and *φ*_ad,*t*_ the probability of existing adult individuals surviving from one year to the next. We consider only the female half of the population (assuming an equal sex-ratio).

The recruitment parameter, *ν*, in the population model (equation (2.2)) may be decomposed into productivity (averaged over breeding and non-breeding individuals) and pre-breeding survival. Willow warblers typically complete one successful breeding attempt per year, although re-nesting may occur if the initial breeding attempt fails very early in the season, and we assume that all females attempt to breed each year, as is typical for short-lived species [33]. We define productivity as the average number of fledged young produced per female per nesting attempt (FPBA), which is the product of brood size and survival at the egg (=hatching success) and chick (=fledging success) stages. These parameters were estimated from data collected as part of the British Trust for Ornithology (BTO) Nest Record Scheme (NRS, [34]; electronic supplementary material, S1.2).

We estimate annual adult survival (*φ*_ad,*t*_) using mark–recapture data collected at sites with constant ringing effort ([35]; electronic supplementary material, S1.3). As very few individuals ringed in their first year (either as pulli or free-flying juveniles) are subsequently recaptured (because of high mortality and dispersal rates), it is not possible to estimate juvenile survival directly. We therefore introduce the parameter, *ρ*, into the recruitment term as a scaling factor to account for this unexplained variation. This parameter quantifies the difference between the observed population size and the measured demographic parameters, which will be owing to factors such as variation in juvenile survival, dispersal, variation in the number of broods per year and the proportion of the population breeding.

As our count of breeding individuals is an index derived from a sampled subset, we use a state-space model to incorporate this observation process. In particular, we assume that the underlying population (*N_t_*) is related in a log-normal fashion to the observed series of counts (*y_t_*), with some degree of observation error (*σ_t_*, [36]):2.4
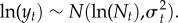


We use a state-space model that combines an underlying system process describing annual population change (equation (2.1)) with an observation process (equation (2.4)) that relates the true population size (*N_t_*) to the observed counts (*y_t_*); productivity is represented as the product of brood size, nest survival (at egg and young stages):2.5
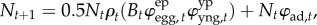
where *B* represents brood size, ep and yp the number of days in which nests contain eggs and nestlings respectively, *φ*_egg,_*φ*_yng_ and *φ*_ad_ are the daily nest survival rates during incubation, brooding and for adults, respectively. Recruitment is multiplied by 0.5 (as only the female part of the population is considered) and, as the actual population size in the first year is unknown (i.e. the observed population trajectory is an index), we arbitrarily set this value to 1000.

We fitted the IPMs using a Bayesian framework, by combining (uninformative) priors on the parameters (adult survival, brood size, egg-stage failure rates, chick-stage failure rate and population size) with their joint probability density function, calculated by multiplying parameter likelihoods together [25,26,36]. Details of prior specification and model fit are provided in the electronic supplementary material, S1.4.

To summarize the posterior distribution of each parameter, we used the Markov chain Monte Carlo (MCMC) algorithm implemented in JAGS v.3.3.0, via the R package rjags [37,38] in R v. 3.1 [39]. We computed 10 chains of 200 000 iterations, of which we discarded the first 100 000 of each as ‘burn-in’ and sampled every 50th, resulting in a posterior MCMC chain of 40 000 parameter estimates. We inspected the traceplots to ensure there was full coverage of the appropriate parameter space and convergence of the MCMC chains was assessed using the Gelman–Rubin statistic 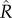 [40]. Convergence was satisfactory for all parameters (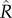 < 1.1).

### (c) Quantifying demographic drivers of population trends

To explore the relationships between demographic rates and population growth, we computed the correlation (over 18 years) between the modelled population growth rate and each demographic rate (annual adult survival, FPBA and its components, and *ρ*) for each MCMC iteration, giving a distribution of 40 000 correlation coefficients. Modelled and observed population growth rates and population indices are strongly positively correlated (electronic supplementary material, figure S2) but modelled values were used as these account for observation error and include imputed values for 2001, the year in which an outbreak of foot and mouth disease in cattle prevented survey work. We report the mean and the 95% credible interval (CRI; represented by the 0.025 and 0.975 quantiles) of this distribution and the probability of a positive correlation (i.e. the proportion of correlation coefficients that are positive). We used the same methodology to explore regional (northwest versus southeast) correlations in demographic rates.

### (d) Demographic characteristics of population growth

To explore the population growth rates associated with differing combinations of survival and productivity, we simulated annual population growth across the full range of estimated annual adult survival rates and productivity produced by the regional IPMs, holding *ρ* constant at the mean level across both regions and years.

### (e) Demographic characteristics of population recovery

**Figure 1.**
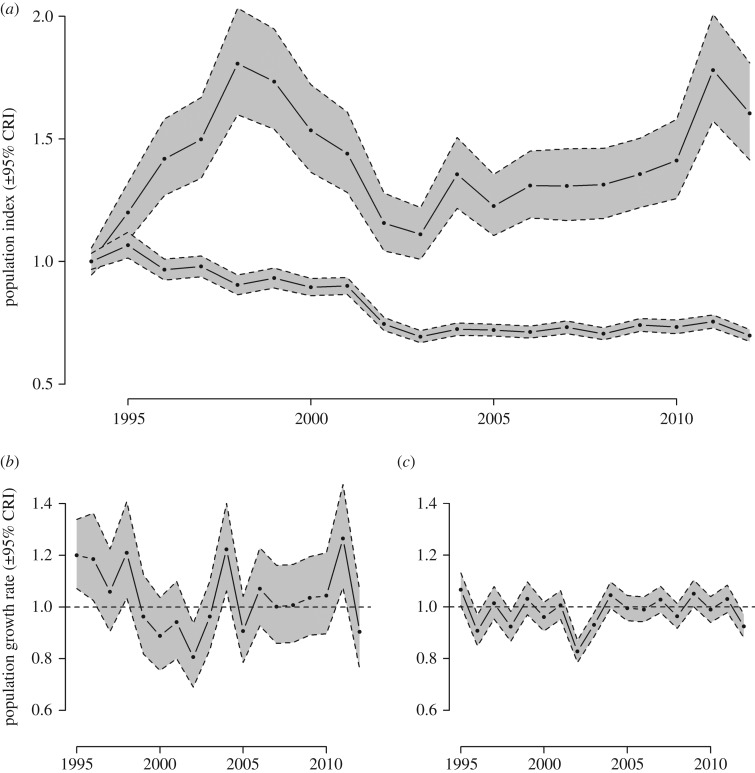
Temporal trends in willow warbler (*a*) population index in the northwest (top line) and southeast (bottom line) regions (indices in both regions set to 1 in 1994), and annual population growth rates in the (*b*) northwest and (*c*) southeast, predicted from IPMs.

Following population declines in both regions between 1999 and 2003, only the northwest population recovered (population index ≥1; figure 1). To explore the demographic changes that could have led to population recovery in the southeast region, we simulated the southeast population index from 2003 onwards (post-decline) using demographic estimates from the northwest region. Two simulations were undertaken; firstly by substituting northwest for southeast survival estimates (termed the survival-substituted trend) and secondly by substituting northwest for southeast FPBA estimates (the productivity-substituted trend). These substitution techniques presume that other demographic variables do not change in response (i.e. no density-dependent impacts on other demographic rates).

## 3. Results

### (a) Regional and temporal variation in population trends

Between 1994 and 2012, willow warbler abundance increased overall by approximately 60% in the northwest region, but declined by approximately 30% in the southeast (figure 1*a*). In both regions, population declines occurred during the late 1990s/early 2000s, however, strongly positive population growth occurred in the northwest region before and after this period (figure 1*b*), while the southeast consistently experienced annual population growth rates close to or below 1 (figure 1*c*).

### (b) Demographic drivers of spatial and temporal variation in willow warbler population trends

**Figure 2.**
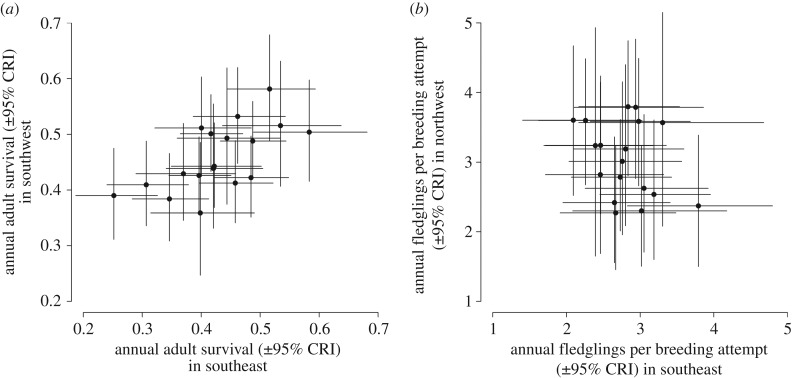
Regional associations between (*a*) annual adult survival rates (mean (over the posterior samples) correlation coefficient = 0.48 (0.22, 0.69 CRIs), probability of positive correlation = 0.99) and (*b*) annual productivity (fledglings per breeding attempt; mean (over the posterior samples) correlation coefficient = −0.17 (−0.50, 0.21), probability of positive correlation = 0.23) in the northwest and the southeast of Britain.

**Figure 3.**
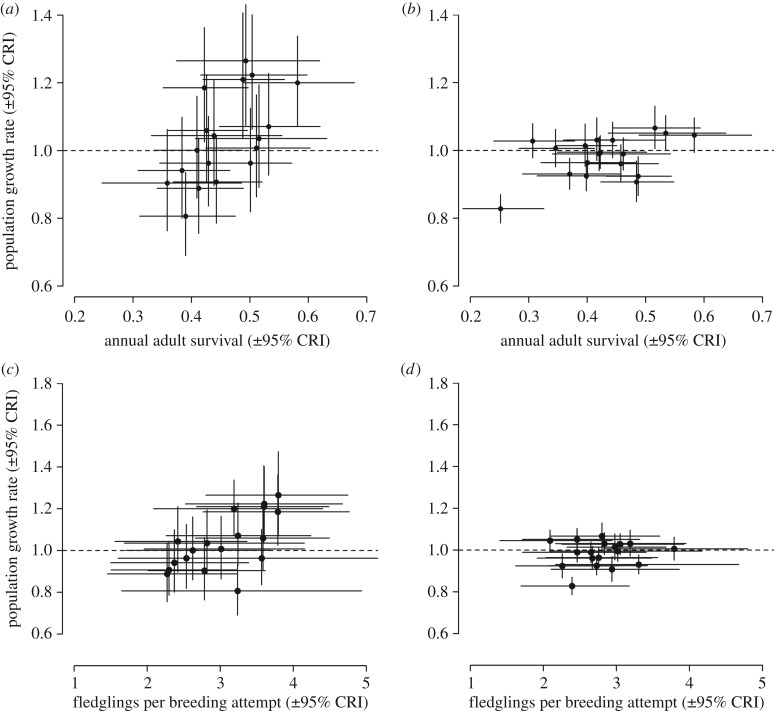
Associations between annual population growth rates and adult survival (top row) and productivity (bottom row) of willow warblers in the northwest (*a,c*) and the southeast (*b,d*) regions. See table 1 for statistical analyses.

Estimates of mean adult annual survival between 1994 and 2012 were very similar in the two regions (table 1), and annual variation in adult survival rates was strongly positively correlated between the regions (figure 2*a*). Annual adult survival rates were also positively correlated with annual variation in population growth rates in both regions (figure 3*a,b*).

**Table 1.** Mean (±95% credible intervals; CRIs) annual variation in demographic rates (*φ*: survival of adults, eggs and chicks over the duration of the incubation and brooding periods, respectively; productivity: fledglings per breeding attempt; *ρ*: unmeasured demographic variation) of willow warblers in the northwest and southeast regions, and their correlation coefficients and probability of a positive correlation with annual population growth rates between 1994 and 2012. (Significant associations are highlighted in italics.)

demographic rate	northwest			southeast		
	mean (95% CRI)	*r* (95% CRI)	*p* (*r* > 0)	mean (95% CRI)	*r* (95% CRI)	*p* (*r* > 0)
*φ*_ad_	0.46 (0.32,0.61)	*0.45 (0.16,0.68)*	*0.99*	0.43 (0.24,0.60)	*0.41 (0.20,0.61)*	*0.99*
brood size	5.78 (5.27,5.99)	−0.09 (−0.36,0.16)	0.29	5.83 (5.37,6.00)	0.31 (−0.05,0.57)	0.93
*φ*_egg_	0.71 (0.38,0.96)	*0.49 (0.17,0.72)*	*0.99*	0.71 (0.49,0.93)	−0.03 (−0.34,0.30)	0.43
*φ*_chick_	0.76 (0.46,0.97)	0.07 (−0.05,0.34)	0.66	0.68 (0.45,0.92)	0.11 (−0.01,0.40)	0.73
productivity	3.04 (1.70,4.56)	*0.53 (0.14,0.80)*	*0.99*	2.80 (1.78,4.11)	0.13 (−0.20,0.43)	0.75
*ρ*	0.39 (0.22,0.60)	0.27 (−0.11,0.56)	0.88	0.41 (0.26,0.57)	0.13 (−0.24,0.46)	0.72

Productivity (FPBA) also did not differ significantly between the regions (overlapping CRIs; table 1), but there was no regional covariation in productivity (figure 2*b*). Productivity was only significantly correlated with population growth rate in the northwest region (table 1 and figure 3*c,d*), and this correlation was primarily driven by variation in nest survival at the egg stage (table 1).

### (c) Demographic characteristics of population growth

**Figure 4.**
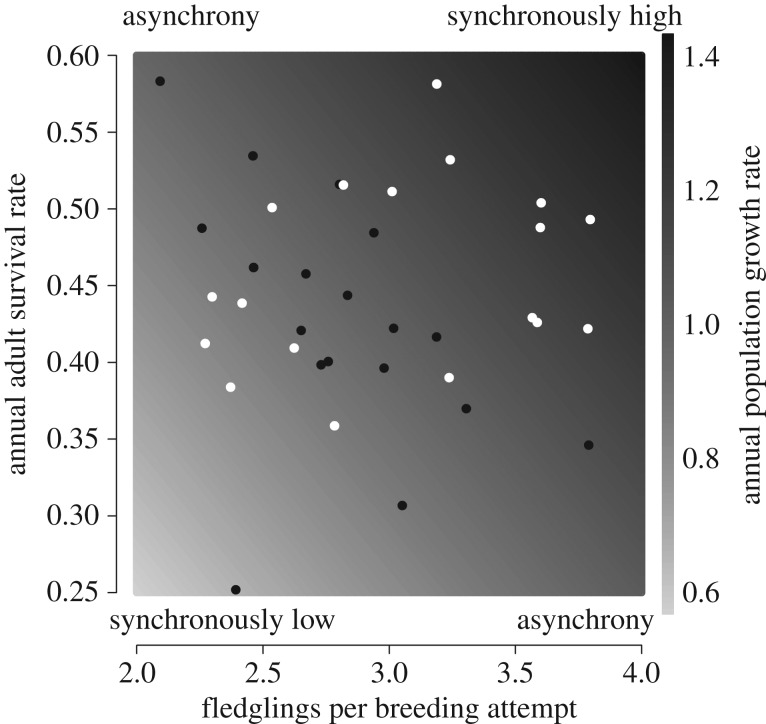
The association between annual adult survival rates and productivity in the northwest (white circles) and southeast (black circles) regions and simulated annual population growth rates, across the observed demographic parameter space (shading).

Simulated annual population growth rates (figure 4, shading) are greatest in years in which both productivity and adult survival rates are high, and these synchronously high years only occurred in the northwest during this time period (figure 4). In the southeast, there have been several years with high survival rates, but none have coincided with high productivity, and the greater frequency of years with low productivity (18 of 19 years with FPBA < 3.5; figure 3*d*) means that years with asynchrony or synchronously low demographic rates were more common in this region (figure 4).

### (d) Demographic characteristics of population recovery

**Figure 5.**
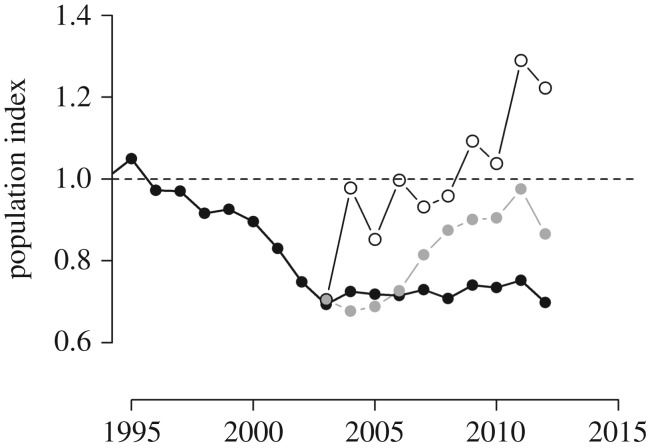
Simulated population trends in the southeast with substituted demographic rates. The predicted trend from the IPM (black circles) is presented with the post-2003 (after the decline) predicted trend had this region experienced either the annual adult survival rates (grey circles) or productivity (fledglings per breeding attempt) levels (open grey circles) achieved in the northwest.

Between 1998 and 2003, large population declines occurred in both regions but, since 2003, the population in the northwest has recovered while the southeast has not (figure 1*a*). Simulated population trajectories for the southeast with substituted demographic rates from the northwest indicate that rapid recovery would have been possible with the levels of productivity achieved in the northwest (figure 5, open circles). While a smaller recovery was also apparent in the survival-substituted trend, full recovery of the population to the levels before the population decline did not occur (figure 5, grey circles). Productivity levels equivalent to those that were achieved in the northwest region after 2003 would therefore have been sufficient to capitalize on the subsequent recovery in survival rates and fully reverse the decline in willow warbler abundance in the southeast.

## 4. Discussion

Many Afro-Palaearctic migratory bird populations are currently declining rapidly across Europe, but the demographic causes of these declines are unknown. Our findings indicate that, while adult survival rates vary greatly between years and contribute substantially to annual population growth rates, differences in productivity have strongly contributed to the divergent population trends in different parts of the breeding range. In the declining southeast population, high productivity was rare (more than 3.5 fledglings per breeding attempt recorded in only 1 out of 19 years) and never coincided with high adult survival. By contrast, high productivity was more common (6 out of 19 years) and coincided with high survival in several years in the increasing northwest population. Thus, while short periods of low adult survival may have initiated population declines in both regions prior to 2003, consistently low productivity has prevented subsequent population recovery in the southeast, despite a nationwide recovery in adult survival rates.

Simulations of population trajectories show that, had the adult survival rates observed in the northwest occurred in the southeast, this would not have been sufficient to fully reverse the population decline in this region (figure 5). Strong regional covariation in annual adult survival rates throughout the period of population declines was apparent (figure 1*a*) suggesting that adult survival rates are influenced by environmental factors operating over large spatial scales, across breeding, passage and/or wintering grounds. Previous studies have linked changes in migrant demography to rainfall in the Sahel [18,19,41] and changes in conditions in similar population bottlenecks, such as the Iberian peninsula, could impact over-wintering and passage conditions for birds from breeding ranges throughout Europe. However, conservation actions designed to influence such large-scale drivers of adult survival rates are likely to be, at best, extremely difficult to design and implement.

By contrast, the levels of productivity observed in the northwest would have been sufficient to fuel population recovery in the southeast, and increasing productivity is likely to be a more achievable target than increasing adult survival, given the availability of relevant policy mechanisms, resources and infrastructure (e.g. protected areas and agri-environment funding) across the European breeding grounds of these species (e.g. [1]). The regional divergence in productivity was primarily a consequence of consistently higher chick survival in the northwest (table 1), suggesting higher rates of chick predation and/or starvation in the southeast. The patchy nature of suitable breeding habitats for migrant passerines in the intensively farmed southeast region is likely to both decrease the availability of food resources and increase vulnerability to nest predators [42].

These IPMs comprise high-quality data on nest success and adult survival, but first year survival cannot be empirically measured at these scales and must therefore be captured by the scaling factor (*ρ*). Variation in population growth rate is well accounted for in the northwest by the available demographic data but less so in the southeast (electronic supplementary material, S1.4), which might imply that juvenile survival, number of broods and/or the proportion of the population breeding [43] are contributing more to annual variation in population change in this region. If lower resource availability is indeed contributing to the low nest survival in the southeast, it may also impact survival during the post-fledging and juvenile stages.

Identifying appropriate actions to reverse population declines can be greatly assisted by knowledge of the demographic processes underpinning population changes [21–23]. In Britain, population declines in willow warblers were initiated by a few consecutive years of poor survival but, in the northwest, these declines were quickly reversed by a recovery of survival rates alongside consistently higher productivity. The consistency of the regional differences in population trends across a suite of long-distance migrants [9,10] suggests that similar demographic processes could be mirrored across these species and, while regional IPMs cannot yet be constructed for these species, they show similar regional differences in productivity (northwest: 3.71 (3.65, 3.79), southeast: 3.44 (3.37, 3.50), *n* = 15 species) but not survival (northwest: 0.44 (0.22, 0.61), southeast: 0.46 (0.34, 0.58), *n* = 5 species) as willow warblers (see the electronic supplementary material, table S5 for species). Identifying and reducing the frequency of conditions associated with low survival is likely to be very difficult to achieve and, on its own, is unlikely to recover populations. By contrast, actions aimed at improving productivity are likely to be substantially more achievable, particularly given the greater availability of relevant funding and infrastructure in European breeding areas, and such actions could benefit many of the declining Afro-Palaearctic migratory species. Increasing the frequency of years of high productivity is likely to buffer populations against years of low survival and to facilitate population growth in years with high survival, and further work is now needed to identify specific actions that can potentially drive improvements in productivity. Actions focused on improving the quality, size and connectivity of breeding habitats (e.g. [44]) may be the most achievable and fruitful means of addressing these rapid and widespread migrant bird declines.

## Supplementary Material

RSPB-2016-0806 revised SOM
