# Supplementary material for: Demographic drivers of decline and recovery in an Afro-Palaearctic migratory bird population
Source: Proc Biol Sci. 2016 Nov 16;283(1842):20161387. doi: 10.1098/rspb.2016.1387 (PMC5124090; doi:10.1098/rspb.2016.1387)
Supplement: RSPB-2016-0806 revised SOM [file rspb20161387supp1.docx]

**Supplementary online material**

**Additional methodology for regional integrated population models**

**1.1 Population size**

Since 1994, the abundance of common breeding bird species within the UK has been monitored as part of the British Trust for Ornithology (BTO)/Royal Society for Protection of birds (RSPB)/Joint Nature Conservation Committee (JNCC) Breeding Bird Survey (BBS) [1,2]. The BBS is a national monitoring survey, however, separate indices can also be produced for different regions of Britain. Here we use separate indices produced from the counties within two broad regions of Britain (north-west and south-east, Fig. S1) in which a suite of migratory bird species are currently showing divergent population trends [3]. Division of this dataset into the two regions follows analyses carried out by [4], which showed areas of stable or increasing population abundance (relative population change > 0) throughout the north and west of Scotland, the north-west of England and the north-west of Wales. Consequently these were grouped into one region (north-west) while populations in the south-west, south-east and north-east mainly showed population declines and were grouped into the south-east region. No abundance data are available for 2001 due to access restrictions following a widespread outbreak of foot-and-mouth disease [5], and we therefore estimated a value for 2001 as the mean of the abundance indices in 2000 and 2002. The indices of population size (*y_t_*) are estimated using a generalised linear model (GLM) in which the (log) number of birds recorded in each site is modelled with a Poisson distribution and a function of two fixed factors: site and year [6]. For each region, the estimated year terms (and associated standard errors) from the GLM provide an index of population size in each year.

**1.2 Productivity**

*Measuring fecundity and nest survival*

Since 1939, the annual breeding performance of a wide range of bird species across Britain has been monitored through the BTO Nest Record Scheme (NRS: for full details see [7]). In the NRS, volunteer observers locate and monitor nests, and record location, date of each visit, and the number and developmental stage of any eggs or young present. This information allows calculation of brood size (the maximum brood size recorded at nests) and the daily probability of nest failure at the incubation and chick-rearing stages. Our estimates of brood size do not take into account partial losses however, evidence suggests that rates of partial loss for willow warblers are extremely low (~2-3%, [8]).

*Modelling productivity*

The annual number of nests included in the calculation of each breeding parameter in each region is reported in Table S1 and correlations between these parameters and sample size and latitude are reported in Tables S4 & S5.

*Brood size*

We modelled brood size using an exponentially-weighted Poisson distribution (EWP), which provides an empirical fit to under-dispersed discrete data [9,10] by incorporating two weighting parameters to ‘squeeze’ the distribution to match the data. [11] found that the fitted weighting parameters did not vary between years, consequently these parameters were fitted as constants i.e. did not vary annually.

*Nest survival*

We followed the Mayfield method [12,13,7] to estimate the probability of nest survival given the number of days a nest was observed to have survived. As the date on which the first egg is laid is not known for most nests, we model average daily nest failure rates within each of the egg incubation and chick-rearing stages. In calculating the probability of survival over the nesting attempt, for willow warbler, we assume eggs are incubated for a fixed period of 14 days and a chick rearing period of 15 days [14]. The probability of survival at the incubation and chick rearing stage was then calculated as (1- daily nest failure rates)^T^, where T = 14 or 15 days respectively. These data were checked for overdispersion by fitting binomial GLMs and calculating the dispersion parameter (the ratio of the residual deviance to the residual degrees of freedom). The dispersion parameter was less than 2 for both the daily egg stage and young stage failure rates in both regions, indicating no overdispersion.

**Table S1:** The annual number of nests contributing to estimates of brood size, and survival rates at the egg-stage (ϕ_egg_) and chick stage (ϕ_yn_) and the annual number of birds contributing to estimates of annual adult survival (ϕ_ad_), in the north-west and south-east of Britain.

| **Region** | **Parameter** | **94** | **95** | **96** | **97** | **98** | **99** | **00** | **01** | **02** | **03** | **04** | **05** | **06** | **07** | **08** | **09** | **10** | **11** | **12** |
| --- | --- | --- | --- | --- | --- | --- | --- | --- | --- | --- | --- | --- | --- | --- | --- | --- | --- | --- | --- | --- |
| North-west | Brood size | 32 | 56 | 78 | 86 | 50 | 63 | 43 | 65 | 39 | 32 | 44 | 60 | 13 | 34 | 19 | 36 | 41 | 86 | 105 |
|  | ϕ_egg_ | 17 | 19 | 36 | 32 | 21 | 21 | 18 | 17 | 14 | 12 | 19 | 29 | 11 | 15 | 9 | 10 | 25 | 41 | 39 |
|  | ϕ_yng_ | 26 | 58 | 61 | 77 | 43 | 46 | 44 | 71 | 33 | 34 | 44 | 51 | 12 | 35 | 13 | 23 | 42 | 68 | 96 |
|  | ϕ_ad_ | 472 | 512 | 602 | 628 | 663 | 575 | 432 | 365 | 346 | 361 | 510 | 403 | 460 | 391 | 224 | 211 | 199 | 223 | 26 |
| South-east | Brood size | 100 | 97 | 124 | 112 | 141 | 191 | 105 | 34 | 44 | 46 | 39 | 53 | 57 | 70 | 56 | 86 | 96 | 127 | 163 |
|  | ϕ_egg_ | 36 | 46 | 58 | 35 | 38 | 32 | 32 | 15 | 11 | 19 | 20 | 17 | 26 | 45 | 22 | 33 | 49 | 53 | 74 |
|  | ϕ_yng_ | 75 | 62 | 73 | 71 | 86 | 123 | 57 | 26 | 37 | 26 | 17 | 31 | 28 | 54 | 43 | 73 | 77 | 97 | 129 |
|  | ϕ_ad_ | 777 | 860 | 911 | 962 | 1046 | 867 | 590 | 390 | 381 | 406 | 519 | 516 | 454 | 436 | 319 | 384 | 401 | 460 | 61 |

**Table S2:** Parameter estimates from Pearson correlations of annual sample sizes and nesting parameters in the north-west and south-east of Britain. (FPBA correlated with the annual sample sizes for the brood size estimates).

| **region** | **variable** | **rho** | **t-value** | **p-value** |
| --- | --- | --- | --- | --- |
| North-west | Brood size | 0.32 | 1.37 | 0.19 |
|  | Daily nest failure rate at egg stage | -0.14 | -0.59 | 0.57 |
|  | Daily nest failure rate at chick stage | -0.27 | -1.14 | 0.27 |
|  | Fledglings per breeding attempt (FPBA) | 0.24 | 0.98 | 0.34 |
| South-east | Brood size | 0.52 | 2.51 | 0.02 |
|  | Daily nest failure rate at egg stage | 0.10 | 0.42 | 0.68 |
|  | Daily nest failure rate at chick stage | -0.05 | -0.20 | 0.84 |
|  | Fledglings per breeding attempt (FPBA) | 0.16 | 0.65 | 0.52 |

**Table S3:** Parameter estimates from general linear models of latitudinal variation in nesting parameters in the north-west and south-east of Britain; t-values are reported for brood sizes (gaussian glms) and z- values are reported for daily nest failure rates (binomial glms).

| **region** | **variable** | **estimate (SE)** | **test-statistic** | **p-value** |
| --- | --- | --- | --- | --- |
| North-west | Brood size | 0.01 (0.03) | 0.41 | 0.68 |
|  | Daily nest failure rate at egg stage | -0.03 (0.08) | -0.38 | 0.71 |
|  | Daily nest failure rate at chick stage | -0.12 (0.07) | -1.80 | 0.07 |
| South-east | Brood size | 0.01 (0.03) | 0.41 | 0.68 |
|  | Daily nest failure rate at egg stage | 0.10 (0.08) | 1.27 | 0.20 |
|  | Daily nest failure rate at chick stage | -0.10 (0.07) | -1.61 | 0.11 |

**1.3 Adult survival**

*Measuring annual adult survival*

We use mark-recapture data from the BTO Constant Effort Site scheme (CES, [15-17]) to estimate annual adult survival probabilities (between breeding seasons). At each CE site, licensed ringers deploy a series of mist nests in the same positions, for the same length of time, during 12 morning and/or evening visits between May and August. We only included years in which sites have been visited more than seven times in the season, had been running for 5 or more years and captured 50 or more individuals (as adults) in total, resulting in 32 and 66 sites in the north-west and south-east regions, respectively. The annual number of individuals caught in each region and used in the calculation of adult survival rates is reported in Table S1.

*Modelling annual adult survival*

In order to gain robust estimates of survival from this large-scale mark recapture dataset, estimates of recapture probabilities are also required. We based our models on the Cormack-Jolly-Seber (CJS) formulation to estimate:

1 – apparent survival probability – the probability that a marked individual alive at sampling occasion t will survive and remain in the population (i.e. not permanently emigrate) between sampling occasion *t* and *t*+1. We allowed survival probabilities to vary between years, but assumed them to be the same across sites within each region.

2 – recapture probability – the probability that a marked individual alive and associated with the population at time *t* will be captured. Because of the constant-effort netting regime, we allow recapture probabilities to vary between sites, but assume they are constant across years.

The CJS model makes a number of assumptions which, if violated, may bias parameter estimates (see [17] for full details). Following [18], we tested goodness of fit using the Release tests implemented in U-Care [19]. Test 3.SR was significant for both the south-east (G^2^ = 192.5, df = 16, p < 0.001) and north-west (G^2^ = 226.7, df = 16, p < 0.001) indicating the presence of a substantial number of transient individuals. In the CES dataset this may be because individuals occupying territories further from the main netting area have a lower likelihood of being recaught, or because individuals migrating through a site in one year are unlikely to be recaught there in subsequent years. We therefore modified the standard CJS model to account for the presence of transient birds by introducing an additional ‘survival period’ in the season of first capture [20,21]. For each bird we insert an additional period after the first capture, indicating whether the bird was recaught subsequently in the same season. The probability of surviving this period can be regarded as the probability that the bird is resident on the site (that is the probability that it will be available for recapture at the same site in subsequent years). Accounting for transients in these models substantially reduces the problems with overdispersion that are typical of capture-recapture data [20] and the sum of the remaining three Release tests indicated no lack of fit for either the south-east (G^2^ = 11.20, df =30, p = 0.99) or the north-west (G^2^ =18.91, df=38, p=0.99) regions. The survival and recapture probabilities for this initial period are assumed constant across all years but vary between sites. Breeding dispersal is also assumed to be absent in these models.

**1.4 Fitting the model**

*Specifying priors.*

For the annual estimates of brood size we specified informative uniform priors (minimum brood size, maximum brood size) as only a small range of values are biologically realistic; for the two weighting parameters of the EWP, we used uniform (0,5) priors. For the annual estimates of mean daily egg and young failure rates we specified uniform (0,1) priors. For the survival probabilities, recapture probabilities and residency probabilities we specified Beta (1,1) priors.

*Assessing model fit*

Modelled population growth rate (eqn 5, including annual variation in *ρ*) was closely correlated with observed annual population growth rate in both regions (Figure S2: north-west, mean (over the posterior samples) correlation coefficient = 0.81 (0.61, 0.93), probability of positive correlation = 1, south-east: mean (over the posterior samples) correlation coefficient = 0.88 (0.71, 0.95), probability of positive correlation = 1). This is unsurprising as *ρ* varies to match the demographic to the observed population change. Observed annual population growth rate (*y_t_/y_t-1_*) was positively correlated with that predicted by the modelled demographic parameters (eqn 5 excluding *ρ*) in the north-west (mean (over the posterior samples) correlation coefficient = 0.50 (0.15, 0.78), probability of positive correlation = 0.99) but not in the south-east (mean (over the posterior samples) correlation coefficient = 0.02 (-0.35, 0.40), probability of positive correlation = 0.54).

**References**

1.

Newson, S.E., Massimino, D., Johnston, A., Baillie, S.R. & Pearce-Higgins, J.W. (2013). Should we account for detectability in population trends? *Bird Study*, 60, 384–390.

2.

Harris, S.J., Risely, K., Massimino, D., Newson, S.E., Eaton, M.A., Musgrove, A.J., *et al.* (2014). The Breeding Bird Survey 2013. *BTO Res. Rep.*, 658.

3.

Balmer, D.E., Gillings, S., Caffery, B.J., Swann, R.L., Downie, I.S. & Fuller, R.J. (2013). *Bird Atlas 2007 - 11: The breeding and wintering birds of Britain and Ireland.* BTO Books, Thetford.

4.

Morrison, C.A., Robinson, R.A., Clark, J.A. & Gill, J.A. (2010). Spatial and temporal variation in population trends in a long-distance migratory bird. *Divers. Distrib.*, 16, 620–627.

5.

Scott, A., Christie, M. & Midmore, P. (2004). Impact of the 2001 foot-and-mouth disease outbreak in Britain: implications for rural studies. *J. Rural Stud.*, 20, 1–14.

6.

# Freeman, S.N., Noble, D.G., Newson, S.E. & Baillie, S.R. (2007). Modelling population changes using data from different surveys: the Common Birds Census and the Breeding Bird Survey. *Bird Study*, 54, 61–72.

# 7.

Crick, H.Q.P., Baillie, S.R. & Leech, D.I. (2003). The UK Nest Record Scheme: its value for science and conservation. *Bird Study*, 50, 254–270.

8.

Simms, E. (1986) *British Warblers*. Collins, London.

9.

Ridout, M.S. & Besbeas, P. (2004). An empirical model for underdispersed count data. *Stat. Modelling*, 4, 77–89.

10.

Robinson, R.A., Baillie, S.R. & King, R. (2012). Population processes in European blackbirds Turdus merula: a state–space approach. *J. Ornithol.*, 152, 419–433.

11.

Robinson, R.A., Morrison, C.A. & Baillie, S.R. (2014). Integrating demographic data: towards a framework for monitoring wildlife populations at large spatial scales. *Methods Ecol. Evol.*, 5, 1361–1372.

12.

Mayfield, H. (1961). Nesting success calculated from exposure. *Wilson Bull.*, 255–261.

13.

Mayfield, H.F. (1975). Suggestions for calculating nest success. *Wilson Bull.*, 456–466.

14.

Ferguson-Lees, J., Castell, R. & Leech, D. (2011). *A Field Guide to Monitoring Nests*. British Trust for Ornithology, Thetford.

15.

Peach, W.J., Buckland, S.T. & Baillie, S.R. (1996). The use of constant effort mist-netting to measure between-year changes in the abundance and productivity of common passerines. *Bird Study*, 43, 142–156.

16.

Peach, W.J., Baillie, S.R. & Balmer, D.E. (1998). Long-term changes in the abundance of passerines in Britain and Ireland as measured by constant effort mist-netting. *Bird Study*, 45, 257–275.

17.

Robinson, R.A., Julliard, R. & Saracco, J.F. (2009). Constant effort: studying avian population processes using standardised ringing. *Ringing Migr.*, 24, 199–204.

18.

Pradel, R., Gimenez, O. and Lebreton, J.D., 2005. Principles and interest of GOF tests for multistate capture–recapture models. Animal Biodiversity and Conservation, 28(2), pp.189-204.

19.

Choquet, R., Lebreton, J.D., Gimenez, O., Reboulet, A.M. and Pradel, R., 2009. U‐CARE: Utilities for performing goodness of fit tests and manipulating CApture–REcapture data. Ecography, 32(6), pp.1071-1074.

20.

Hines, J.E., Kendall, W.L., and Nichols, J.D. (2003). On the use of the robust design with transient capture-recapture models. *Auk*, 120, 1151–1158.

21.

Rosenberg, D. K., D. F. DeSante, K. S. McKelvey, and J. E. Hines. 1999. Monitoring survival rates of Swainson’s Thrush *Catharus ustulatus* at multiple spatial scales. *Bird Study* 46(Supplement):198–208

**Table S4:** Annual estimates (CRIs) of the population index, population growth rate (R), brood size, and survival rates at the egg-stage (ϕ_egg_) and chick stage (ϕ_yn_), fledglings per breeding attempt (FPBA) and annual adult survival (ϕ_ad_), in the north-west and south-east of Britain. Estimates of ϕ_ad_, rho and R run from 1994-1995 to 2011-2012.

| **Region** | **Year** | **Population index** | **R** | **Brood size** | **ϕ_egg_** | **ϕ_chick_** | **FPBA** | **ϕ_ad_** | **rho** |
| --- | --- | --- | --- | --- | --- | --- | --- | --- | --- |
| **North-west** | 1994 | 1.00  (0.95,1.06) | 1.20  (1.07,1.34) | 5.77  (5.45,5.99) | 0.64  (0.43,0.86) | 0.85  (0.68,0.86) | 3.19  (2.09,4.40) | 0.58 (0.49,0.68) | 0.40 (0.27,0.57) |
|  | 1995 | 1.19  (1.08,1.32) | 1.19  (1.03,1.36) | 5.94  (5.80,6.00) | 0.79  (0.60,0.95) | 0.80  (0.37,0.92) | 3.79  (2.77,4.77) | 0.42 (0.35,0.50) | 0.41  (0.29,0.56) |
|  | 1996 | 1.42  (1.27,1.58) | 1.06  (0.91,1.22) | 5.81  (5.58,5.98) | 0.84  (0.67,0.96) | 0.73  (0.60,0.87) | 3.59  (2.66,4.49) | 0.43 (0.36,0.50) | 0.36  (0.25,0.49) |
|  | 1997 | 1.50  (1.34,1.67) | 1.21  (1.03,1.41) | 5.86  (5.66,5.99) | 0.78  (0.60,0.93) | 0.78  (0.67,0.90) | 3.60  (2.67,4.49) | 0.49 (0.42,0.56) | 0.41  (0.29,0.55) |
|  | 1998 | 1.81  (1.60,2.03) | 0.96  (0.82,1.13) | 5.86  (5.62,5.99) | 0.63  (0.42,0.84) | 0.68  (0.52,0.86) | 2.54  (1.60,3.61) | 0.5 (0.43,0.57) | 0.37  (0.23,0.55) |
|  | 1999 | 1.73  (1.54,1.95) | 0.89  (0.75,1.04) | 5.90  (5.70,6.00) | 0.46  (0.30,0.66) | 0.83  (0.70,0.94) | 2.27  (1.45,3.24) | 0.41 (0.34,0.49) | 0.43  (0.28,0.61) |
|  | 2000 | 1.54  (1.36,1.72) | 0.94  (0.80,1.10) | 5.86  (5.62,5.99) | 0.45  (0.29,0.65) | 0.89  (0.77,0.98) | 2.37  (1.50,3.39) | 0.38  (0.31,0.47) | 0.48  (0.33,0.70) |
|  | 2001 | 1.44  (1.28,1.61) | 0.81  (0.69,0.94) | 5.93  (5.77,6.00) | 0.66  (0.34,0.98) | 0.80  (0.67,0.93) | 3.24  (1.65,4.93) | 0.39  (0.31,0.47) | 0.28 (0.14,0.47) |
|  | 2002 | 1.16  (1.04,1.28) | 0.96  (0.84,1.10) | 5.90  (5.69,6.00) | 0.64  (0.38,0.93) | 0.92  (0.77,1.0) | 3.57  (2.08,5.21) | 0.43  (0.35,0.52) | 0.32  (0.19,0.49) |
|  | 2003 | 1.11  (1.01,1.22) | 1.22  (1.06,1.40) | 5.63  (5.27,5.95) | 0.83  (0.32,0.98) | 0.76  (0.59,0.93) | 3.60  (2.52,4.67) | 0.50  (0.42,0.60) | 0.41  ((0.28,0.56) |
|  | 2004 | 1.36  (1.22,1.51) | 0.91  (0.79,1.04) | 5.89  (5.68,6.00) | 0.62  (0.42,0.82) | 0.62  (0.48,0.81) | 2.30  (1.50,3.23) | 0.44  (0.37,0.52) | 0.41  (0.27,0.59) |
|  | 2005 | 1.23  (1.11,1.36) | 1.07  (0.93,1.23) | 5.45  (5.16,5.73) | 0.70  (0.50,0.88) | 0.84  (0.71,0.96) | 3.24  (2.26,4.24) | 0.53  (0.45,0.62) | 0.34  (0.22,0.49) |
|  | 2006 | 1.31  (1.18,1.45) | 1.00  (0.86,1.16) | 5.82  (5.45,5.99) | 0.67  (0.47,0.86) | 0.66  (0.48,0.89) | 2.62  (1.71,3.69) | 0.41  (0.34,0.49) | 0.46  (0.32,0.66) |
|  | 2007 | 1.31  (1.17,1.46) | 1.01  (0.86,1.16) | 5.58  (5.22,5.91) | 0.75  (0.53,0.94) | 0.71  (0.53,0.89) | 3.01  (1.96,4.16) | 0.51  (0.42,0.60) | 0.34  (0.21,0.49) |
|  | 2008 | 1.31  (1.18,1.46) | 1.04  (0.89,1.19) | 5.59  (5.14,5.95) | 0.67  (0.42,0.91) | 0.74  (0.50,0.96) | 2.82  (1.69,4.15) | 0.52  (0.41,0.63) | 0.38  (0.23,0.57) |
|  | 2009 | 1.36  (1.22,1.50) | 1.04  (0.90,1.21) | 5.44  (5.02,5.83) | 0.90  (0.70,0.99) | 0.48  (0.35,0.69) | 2.42  (1.55,3.36) | 0.44  (0.33,0.55) | 0.51  (0.35,0.73) |
|  | 2010 | 1.41  (1.26,1.58) | 1.27  (1.07,1.47) | 5.92  (5.75,6.00) | 0.86  (0.70,0.97) | 0.74  (0.59,0.90) | 3.80  (2.80,4.75) | 0.49  (0.37,0.62) | 0.41  (0.29,0.56) |
|  | 2011 | 1.78  (1.57,2.01) | 0.90  (0.76,1.06) | 5.77  (5.54,5.97) | 0.64  (0.64,0.80) | 0.74  (0.63,0.87) | 2.78  (2.01,3.61) | 0.36  (0.25,0.49) | 0.40  (0.27,0.55) |
|  | 2012 | 1.60  (1.41,1.81) |  | 5.83  (5.62,5.98) | 0.81  (0.65,0.93) | 0.77  (0.67,0.88) |  |  |  |
| **South-east** | 1994 | 1  (0.97,1.03) | 1.07  (1.00,1.13) | 5.93  (5.79,6.00) | 0.88  (0.74,0.97) | 0.55  (0.43,0.69) | 2.80  (2.10,3.59) | 0.52  (0.44,0.59) | 0.40  (0.29,0.52) |
|  | 1995 | 1.07  (1.01,1.12) | 0.91  (0.85,0.97) | 5.90  (5.72,6.00) | 0.78  (0.62,0.91) | 0.65  (0.50,0.81) | 2.94  (2.10,3.86) | 0.48  (0.42,0.55) | 0.30  (0.20,0.42) |
|  | 1996 | 0.97  (0.92,1.01) | 1.01  (0.95,1.08) | 5.72  (5.51,5.92) | 0.71  (0.58,0.83) | 0.74  (0.62,0.86) | 2.98  (2.32,3.68) | 0.40  (0.35,0.45) | 0.42  (0.33,0.54) |
|  | 1997 | 0.98  (0.94,1.02) | 0.92  (0.87,0.98) | 5.88  (5.71,5.99) | 0.68  (0.51,0.85) | 0.58  ((0.45,0.73) | 2.26  (1.62,3.03) | 0.49  (0.43,0.54) | 0.40  (0.28,0.53) |
|  | 1998 | 0.90  (0.86,0.94) | 1.03  (0.97,1.10) | 5.94  (5.82,6.00) | 0.76  (0.62,0.89) | 0.72  (0.60,0.83) | 3.19  (2.45,3.95) | 0.42  (0.36,0.47) | 0.39  (0.30,0.51) |
|  | 1999 | 0.93  (0.89,0.97) | 0.96  (0.91,1.02) | 5.97  (5.91,6.00) | 0.68  (0.49,0.86) | 0.67  (0.57,0.78) | 2.67  (1.91,3.49) | 0.46  (0.40,0.52) | 0.39  (0.28,0.52) |
|  | 2000 | 0.90  (0.86,0.93) | 1.01  (0.95,1.06) | 5.93  (5.80,6.00) | 0.71  (0.54,0.89) | 0.89  (0.77,0.98) | 3.79  (2.82,4.79) | 0.35  (0.28,0.41) | 0.36  (0.27,0.47) |
|  | 2001 | 0.90  (0.87,0.94) | 0.83  (0.79,0.87) | 5.39  (4.97,5.78) | 0.81  (0.62,0.95) | 0.57  (0.41,0.74) | 2.39  (1.69,3.18) | 0.25  (0.19,0.33) | 0.49  (0.36,0.66) |
|  | 2002 | 0.75  (0.72,0.77) | 0.93  (0.89,0.98) | 5.82  (5.53,5.99) | 0.68  (0.45,0.94) | 0.83  (0.65,0.96) | 3.31  (2.17,4.68) | 0.37  (0.29,0.46) | 0.35  (0.24,0.50) |
|  | 2003 | 0.69  (0.67,0.72) | 1.05  (0.99,1.10) | 5.85  (5.58,5.99) | 0.61  (0.43,0.81) | 0.60  (0.42,0.79) | 2.09  (1.40,2.94) | 0.58  (0.49,0.68) | 0.45  (0.31,0.62) |
|  | 2004 | 0.72  (0.70,0.75) | 0.99  (0.95,1.04) | 5.85  (5.58,5.99) | 0.80  (0.60,0.95) | 0.66  (0.46,0.87) | 3.02  (2.08,4.17) | 0.42  (0.35,0.50) | 0.39  (0.27,0.54) |
|  | 2005 | 0.72  (0.70,0.74) | 0.99  (0.94,1.04) | 5.76  (5.48,5.98) | 0.69  (0.49,0.89) | 0.64  (0.47,0.81) | 2.46  (1.71,3.36) | 0.46  (0.39,0.54) | 0.44  (0.31,0.59) |
|  | 2006 | 0.71  (0.69,0.74 | 1.03  (0.98,1.08) | 5.87  (5.64,5.99) | 0.70  (0.54,0.86) | 0.75  (0.59,0.89) | 3.05  (2.25,3.92) | 0.31  (0.24,0.38) | 0.48  (0.36,0.63) |
|  | 2007 | 0.73  (0.71,0.76) | 0.96  (0.92,1.01) | 5.85  (5.64,5.99) | 0.61  (0.46,0.77) | 0.78  (0.64,0.90) | 2.76  (2.03,3.56) | 0.40  (0.32,0.49) | 0.41  (0.31,0.55) |
|  | 2008 | 0.71  (0.68,0.73) | 1.05  (1.00,1.10) | 5.77  (5.49,5.98) | 0.58  (0.41,0.76) | 0.75  (0.59,0.89) | 2.46  (1.71,3.32) | 0.53  (0.44,0.64) | 0.43  (0.31,0.58) |
|  | 2009 | 0.74  (0.72,0.77) | 0.99  (0.94,1.04) | 5.83  (5.61,5.99) | 0.71  (0.55,0.86) | 0.66  (0.53,0.79) | 2.65  (1.95,3.41( | 0.42  (0.34,0.50) | 0.43  (0.32,0.57) |
|  | 2010 | 0.73  (0.71,0.76) | 1.03  (0.98,1.08) | 5.74  (5.51,5.95) | 0.73  (0.60,0.85) | 0.69  (0.56,0.81) | 2.84  (2.17,3.53) | 0.44  (0.36,0.53) | 0.42  (0.32,0.54) |
|  | 2011 | 0.76  (0.73,0.78) | 0.92  (0.88,0.97) | 5.95  (5.83,6.00) | 0.62  (0.49,0.76) | 0.75  (0.64,0.85) | 2.73  (2.07,3.43) | 0.40  (0.31,0.49) | 0.39  (0.29,0.51) |
|  | 2012 | 0.70  (0.67,0.72) |  | 5.90  (5.75,5.99) | 0.65  (0.52,0.77) | 0.69  (0.58,0.79) |  |  |  |

**Table S5:** Species included in the analysis of regional variation in demography. Species in bold indicate species used for both the analysis of productivity and survival rates, otherwise species were only included in the analysis of productivity.

| **Species** |
| --- |
| Sand martin |
| Yellow wagtail |
| Redstart |
| Wheatear |
| **Sedge warbler** |
| Lesser whitethroat |
| **Whitethroat** |
| Tree pipit |
| Whinchat |
| **Reed warbler** |
| **Garden warbler** |
| Wood warbler |
| **Willow warbler** |
| Spotted flycatcher |
| Pied flycatcher |

**Figure S1:** Areas of Britain included within north-west (white) region in which willow warbler populations are stable or increasing and south-east (grey) region in which willow warbler populations are declining (Morrison *et al.* 2010).

**
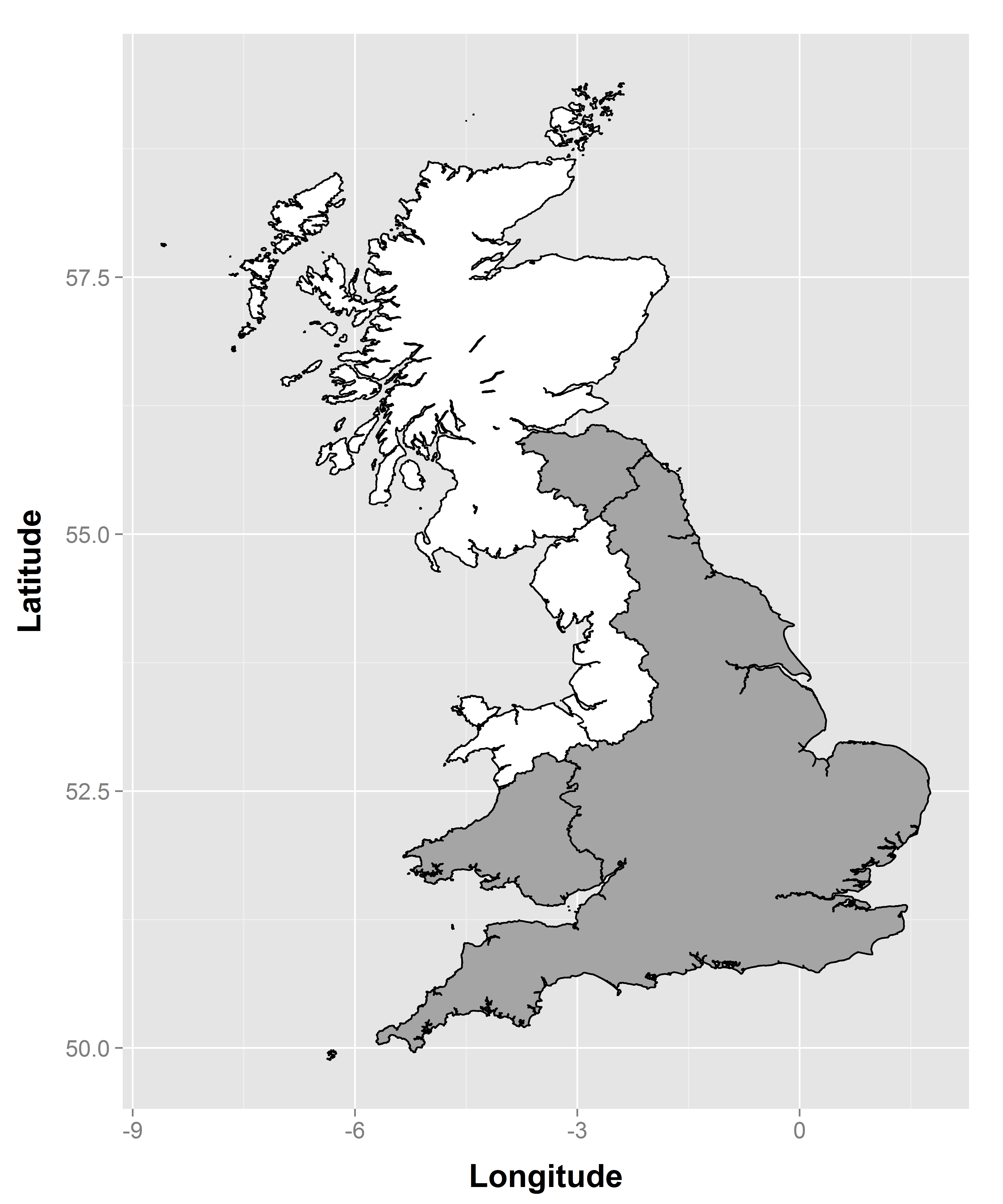
**

**Figure S2:** Relationship between the predicted and observed population growth rates (top row, north-west, mean (over the posterior samples) correlation coefficient = 0.81 (0.61, 0.93), probability of positive correlation = 1, south-east: mean (over the posterior samples) correlation coefficient = 0.88 (0.71, 0.95), probability of positive correlation = 1) and population indices (bottom row, north-west, mean (over the posterior samples) correlation coefficient = 0.94 (0.90, 0.97), probability of positive correlation = 1, south-east: mean (over the posterior samples) correlation coefficient = 0.99 (0.98, 1.0), probability of positive correlation = 1).

**
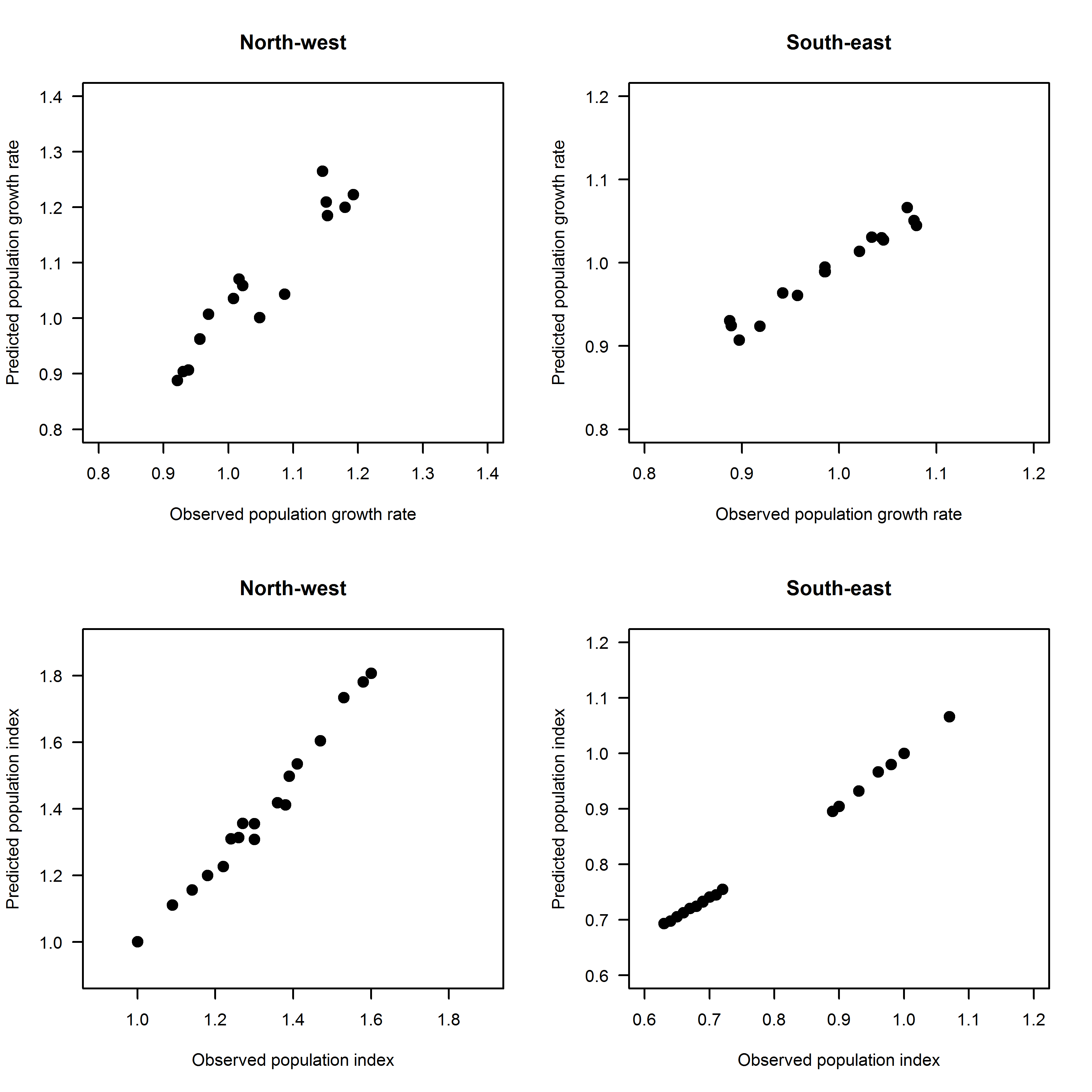
**

**Code S1:** JAGS code to fit the population model

model{

####POPULATION MODEL: SYSTEM PROCESS

# Define the prior for 'number of broods' parameter

nb_mean ~ dunif(0,10)

nb_tau ~ dgamma(0.01,0.01)

# because recursive need to set population in first year

N1[1] <- 0

Na1 ~ dpois(1000)

Na[1] <- round(Na1)

# The system process: a simple annual stochastic demographic model

for ( i in 1:nyears-1){

# rho has to be positive

rho[i] ~ dnorm(nb_mean,nb_tau)

# Number of birds fledged per attempt

Nfledge[i] <- bs_mean[i] * pow((1-dfail_e[i]),14 ) * pow((1-dfail_y[i]), 15)

# Number of recruits into breeding population the following year

Nr[i] <- Na[i] * rho[i] * 0.5*Nfledge[i]

# Number of adults surviving

Ns[i] ~ dbin(phi[i],Na[i])

}

# for remaining years add recruits and surviving adults

for ( i in 2:nyears){

N1[i] ~ dpois(Nr[i-1])

Na[i] <- N1[i] + Ns[i-1]

}

# Calculate Population Growth Rate

for ( i in 1:nyears-1){

R[i] <- Na[i+1] / Na[i]

}

#### POPULATION MODEL: OBSERVATION PROCESS

# Observation process: adults are a normal sample of those existing

# with sigma based on MLE parameters corrected according to King et al (2008)

# y[i] and yse[i] are on log scale so need to transform Na

yse[1]~dunif(0,50)

yse[8]~dunif(0,50) # 2001 is foot-and-mouth year

for ( i in 1:nyears) { ###removed nyears+1 as added on 1 to nyears

obs_tau[i] <- 1 / pow(yse[i],2)

lnNa[i] <- log(Na[i])

y[i] ~ dnorm(lnNa[i], obs_tau[i])

}

#### DEMOGRAPHIC PROCESSES

#### SURVIVAL RATES: from CES RINGING DATA

#### Priors

for (d in 1:nyears-1){

phi[d]<-alpha[d] ####Survival

}

####Survival

for (i in 1:nyears-1){

alpha[i]~dunif(0,1) }

# Prior for mean annual recapture and residency

for(s in 1:n.sites){

p.s[s] ~ dbeta(1, 1)

res[s] ~ dbeta(1,1)

beta.p[s] ~ dnorm(0, 0.001)I(-10, 10)

}

#####within season recapture probability

for (i in 1:n.individuals){ # recapture within first season depends on visit of first capture

logit(p0[i]) <- beta.p[site[i]] * visit[i] #### visit = 12 (total no of visits) - visit number

}

# Likelihood

for (i in 1:n.individuals){

# first capture, so this = 1 by definition

z[i, ff[i]] <- 1

# First Year - within season survival(=residency)

z[i,(ff[i]+1)] ~ dbern(res[site[i]]) # State process

# Observation process, within season recapture probability in first year

yy[i,(ff[i]+1)] ~ dbern(mu0[i, ff[i]])

mu0[i, ff[i]] <- z[i, (ff[i]+1)] * p0[i]

# Now subsequent years - between year survival

for (t in ff[i]+2:last.year[i]+1){

# State process

z[i, t] ~ dbern(mu1[i, t-2])

mu1[i, t-2] <- phi[t-2] * z[i, t-1]

# Observation process

yy[i, t] ~ dbern(mu2[i, t-2])

mu2[i, t-2] <- p.s[site[i]] * z[i, t]

} #t

} #i

## PRODUCTIVITY: BROOD SIZES from RINGING DATA

## Define the priors

for (i in 1:nyears){

# Use an informative prior for brood size since we have a good idea of what they might be

bs_mean[i] ~ dunif(1, 6)

}

# two weighting parameters for exponential Poisson

Beta1 ~ dunif(0,5)

Beta2 ~ dunif(0,5)

## Calculate the likelihood

# first calculate brood-size frequency

for ( i in 1:nyears){

for ( k in min_brood:max_brood ){

# use step() to identify whether greater or less than mean brood size

ind[i,k] <- step(k-bs_mean[k])

w_k[i,k] <- ind[i,k] * exp(-1*Beta2*(k-bs_mean[i])) + (1-ind[i,k]) * exp(-1*Beta1*(bs_mean[i]-k))

w[i,k] <- (exp(-1*bs_mean[i])*pow(bs_mean[i],k)*w_k[i,k])/exp(logfact(k))

}

wcap[i]<-sum(w[i,])

}

# now sample from it

for ( i in 1:nyears ){

for ( k in min_brood:max_brood ){

f[i,k] <- w[i,k] / wcap[i]

bsize[i,k] ~ dbin(f[i,k],ncases[i])

}

}

## PRODUCTIVITY: NEST FAILURE RATES from NEST RECORD DATA

## Define the priors

for ( i in 1:nyears ) {

dfail_e[i] ~ dbeta(1,1)

dfail_y[i] ~ dbeta(1,1)

}

## Calculate the likelihoods: note these are daily failure rates

for ( i in 1:erec ){

edays[i] <- egg_ed[i] * etrial[i]

efail[i] ~ dbin(dfail_e[eggyear[i]], edays[i])

}

for ( i in 1:yrec ){

ydays[i] <- yng_ed[i] * ytrial[i]

yfail[i] ~ dbin(dfail_y[yngyear[i]], ydays[i])

}

}**Data S2:** R code to initialize and perform the MCMC updates as parallel chains.

require(parallel)

require(boot)

require(rjags)

jags.data <- source("JAGSdata.txt")[[1]]

###function create a matrix with information about known latent state z

known.state.cjs <- function(ch){

state <- ch

for (i in 1:dim(ch)[1]){

n1 <- min(which(ch[i,]==1)) ###min 1

n2 <- max(which(ch[i,]==1)) ###max 1

state[i,n1:n2] <- 1 ###all inbetween min and max = 1

state[i,n1] <- NA #####first cap =1

}

state[state==0] <- NA ###if not 1 NA

return(state)

}

####function to create a matrix of initial values for latent state z

cjs.init.z <- function(ch,f){

for (i in 1:dim(ch)[1]){

if (sum(ch[i,])==1) next

n2 <- max(which(ch[i,]==1))

ch[i,f[i]:n2] <- NA

}

for (i in 1:dim(ch)[1]){

ch[i,1:f[i]] <- NA

}

return(ch)

}

jags.inits <- function(){

list(

### SURVIVAL (phi), RECOVERY (p) and residency (res) PROBABILITIES

z=cjs.init.z(CH,f),

phi=rep(0.3,nyears-1),

p.s=rep(0.4,n.sites),

beta.p=rep(0.4,n.sites),

res=runif(n.sites,0.6),

### PRODUCTIVITY: BROOD SIZES

dfail_e = rep(0.03,nyears),

dfail_y = rep(0.03,nyears),

### PRODUCTIVITY: NEST FAILURE RATES

mean.ef=runif(ntp,0,1),

mean.yf=runif(ntp,0,1),

### POPULATION: SYSTEM PROCESS

nb_mean=2.0,

nb_tau=dgamma(0.01,0.01),

rho_mean = 2.0,

rho = rep(1.0, nyears-1)

)

}

run_jags_model <- function(...){

library(rjags)

library(boot)

load.module('lecuyer')

n.burnin <- 100000

n.samples <- 200000

n.thin <- 50

model <- jags.model(file="JAGS model.txt", data = jags.data, inits = jags.inits, n.chains = 2)

update(model, n.burnin)

coda.samples(model, parameters, n.samples, n.thin)

}

mc <- 5

cl <- makeCluster(mc) # set up a 'cluster' with mc cores

clusterSetRNGStream(cl, 123) # initialise the random number generator

jags1 <- do.call(c, parLapply(cl, seq_len(mc), run_jags_model) ) # run the model on each core

stopCluster(cl) # close the cluster

save.image("Wilwa.RData")
